# External-beam partial breast irradiation in a supine versus prone position after breast-conserving surgery for Chinese breast cancer patients

**DOI:** 10.1038/s41598-018-33741-z

**Published:** 2018-10-18

**Authors:** Ting Yu, Min Xu, Tao Sun, Qian Shao, YingJie Zhang, XiJun Liu, FengXiang Li, Wei Wang, Jian Bin Li

**Affiliations:** 1 School of Medicine and Life Sciences, University of Jinan-Shandong Academy of Medical Sciences, Jinan, Shandong province China; 2 Department of Radiation Oncology, Shandong Cancer Hospital Affiliated to Shandong University, Shandong Academy of Medical Sciences, Jinan, Shandong province China; 3 Department of Radiophysics, Shandong Cancer Hospital Affiliated to Shandong University, Shandong Academy of Medical Sciences, Jinan, Shandong province China

## Abstract

To investigate the differences in target volumes and dosimetric parameters between the supine and prone positions for external-beam partial breast irradiation (EB-PBI) after breast-conserving surgery (BCS) for Chinese breast cancer patients, thirty breast cancer patients who underwent three-dimensional conformal radiation therapy (3DCRT) EB-PBI after BCS were enrolled. Supine and prone scan sets were acquired during free breathing for all patients. Target volumes and organs at risk (OARs) including the heart, ipsilateral lung and bilateral breast were contoured by the same radiation oncologist. For each patient, supine and prone EB-PBI plans were generated based on the same planning criteria. The clinical target volume (CTV) and planning target volume (PTV) in the prone position were significantly greater than those in the supine position (*P* = 0.003, 0.004, respectively). A 0.95 Gy reduction in the mean dose (*D*_mean_) to the heart (*P* = 0.000) was apparent in the supine position compared to the prone position. The *D*_mean_ to the ipsilateral lung was significantly lower in the prone position than in the supine position (1.59 Gy vs. 1.72 Gy, *P* = 0.029). Therefore, for Chinese breast cancer patients, carrying out 3DCRT EB-PBI in the prone position during free breathing is feasible.

## Introduction

Breast-conserving therapy (BCT) has become a standard of care in the treatment of early-stage breast cancer, and radiotherapy after breast-conserving surgery (BCS) plays an important role in BCT. Breast irradiation not only results in a reduction in local and regional recurrence after BCS but also decreases the death rate effectively^1^. For adjuvant radiotherapy after BCS, supine positioning is still the most common approach and has multiple advantages, such as methodological simplicity, comfort and accurate and reproducible positioning^2^. Due to the deformability and softness of the breast, during simulation and treatment in the supine position, the breast stretches over the chest wall, especially in patients with large and pendulous glands. Thus, the ipsilateral lung (IPSL) and heart are inevitably irradiated during radiotherapy, and the region of the skin fold is increased. Hence, the radiotherapeutic toxicity, including radiation-induced lung and heart toxicity and skin injury, are unavoidable^3,4^. Additionally, the occurrence rate of pulmonary and cardiovascular events increases after supine radiotherapy regimens along with the prolongation of time^5,6^.

In 1994, Merchant *et al*.^7^ became the first to describe the prone breast technique after BCS in detail. The authors reported that prone breast irradiation reduced the radiation exposure dose to the lung and relieved the acute reaction of the skin, particularly in patients with large and pendulous glands. At present, several dosimetric studies^8,9^ have reported that, compared to conventional supine breast irradiation, the mean dose (*D*_mean_) and volumes that receive equal or more than 20 Gy to the lung are decreased remarkably in prone breast irradiation after BCS. Moreover, this characteristic has nothing to do with the breast target volumes^10,11^. Meanwhile, the prone positioning of patients during whole breast radiation (WBI) can also improve dose homogeneity and reduce the high dose distribution in the target^12,13^. Based on these reports, prone breast irradiation has received a great deal of attention.

However, the present comparative studies on supine and prone breast irradiation after BCS are mainly aimed at WBI. Over the past several years, partial breast irradiation (PBI) has become an option for radiation therapy in early-stage breast cancer patients with low risk after BCS. Prone PBI planning for breast cancer patients has been reported^10^. Furthermore, external-beam PBI (EB-PBI) is an important approach of PBI. However, compared to supine EB-PBI, the advantages of the specified dosimetric parameters for the target volume and organs at risk (OARs) for prone EB-PBI planning have not been established. Therefore, in this study, we compared the EB-PBI treatment plans in the prone versus supine positions, and our emphasis was on investigating the differences in the volumetric and dosimetric parameters for EB-PBI in the two positions.

## Methods

### Patient selection

Breast cancer patients who were suitable for EB-PBI after BCS were enrolled in this study between July 2016 and April 2017. All patients underwent axial 3DCT simulation scanning in the supine and prone position for treatment planning during free breathing. Patients with oncoplastic BCS were excluded from the trial, and all the enrolled patients had ≥5 surgical clips fixed to the central bottom and lateral edges of the surgical cavity to mark the tumour bed boundaries. Postoperative pathological stage was pT1N0M0 in all patients. None of the patients had chronic lung diseases, and all exhibited normal arm movement after surgery. Written informed consent forms were obtained from all patients, and the study was approved by the institutional research ethics board of the Shandong Tumour Hospital Ethics Committee. The research was performed in accordance with revelant regulations and all patients in our research voluntarily joined this study with informed consents.

### CT simulation

**Figure 1:**
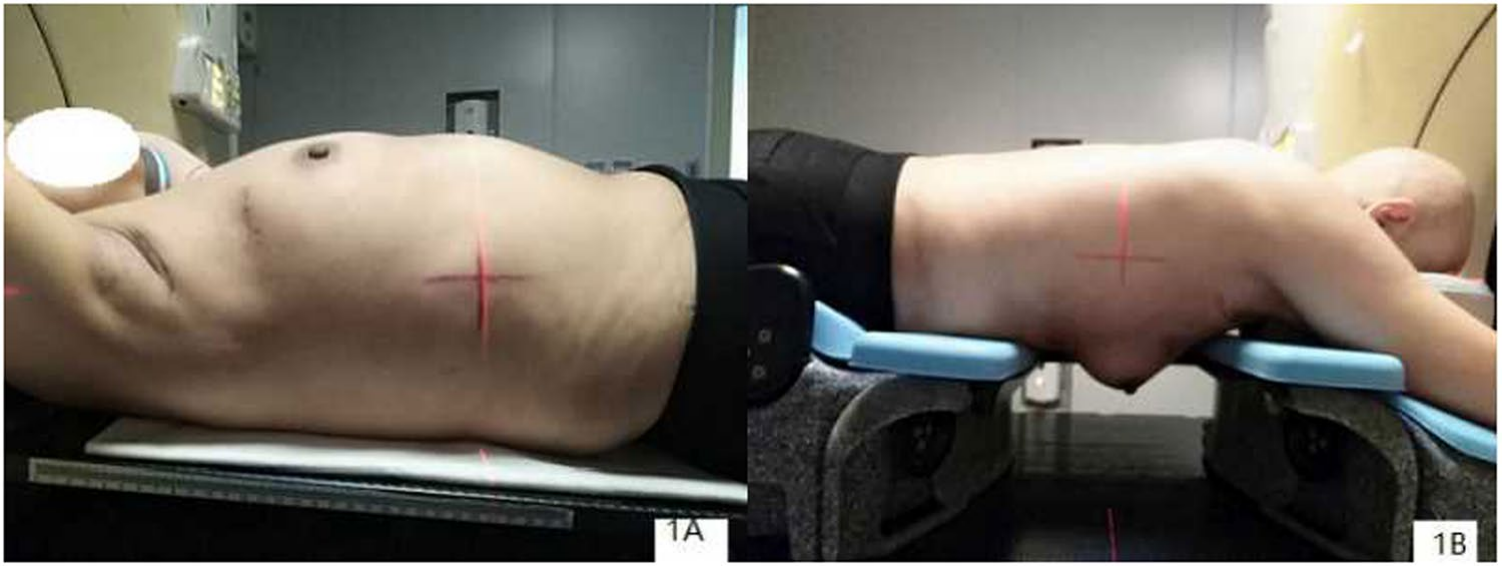
The pictures of CT simulation in supine and prone position (**A**. supine position; **B**. prone position).

During simulation, all patients were scanned in both the supine and prone positions. For each patient, the 3DCT data sets in the different positions were acquired on a 16-slice CT scanner (Philips Brilliance Bores CT, Netherlands) during free breathing. For the supine position, the patients were immobilized on a breast board (CIVCO - MT350N) with no degree incline using an arm support (with both arms above the head to expose the breast adequately) and a knee support (Fig. 1A). Afterwards, the patients were placed in the prone position on a dedicated treatment board (CIVCO Horizon^TM^ Prone Breast Bracket- MTHPBB01) with no degree incline using an arm support (with both arms above the head). The board contained an open aperture on one side to allow for the ipsilateral breast to hang freely away from the chest wall (Fig. 1B). All CT images were reconstructed using a thickness of 3 mm and then transferred to the Eclipse treatment planning system (Eclipse 13.5, Varian Medical Systems, Palo Alto, CA, USA) for target and OAR delineation and to formulate treatment plans.

### Target definition

All structures were delineated by the same radiation oncologist who had implemented the breast cancer irradiation plan; the oncologist had over 5 years of experience in radiotherapy. The gross target volume (GTV) was delineated based only on the surgical clips. The clinical target volume (CTV) consisted of the GTV plus a 10-mm margin, and the CTV was limited to 5 mm from the skin surface and gland-pectorales interface. The planning target volume (PTV) was contoured and expanded equably by a 5-mm margin from the CTV and was limited to 5 mm from the skin surface and lung-chest wall interface. The OARs, including the heart, IPSL and bilateral breast, were contoured over the full range of images obtained. Both the prone and supine setups were determined using the same clinical criteria.

### Treatment planning

**Figure 2:**
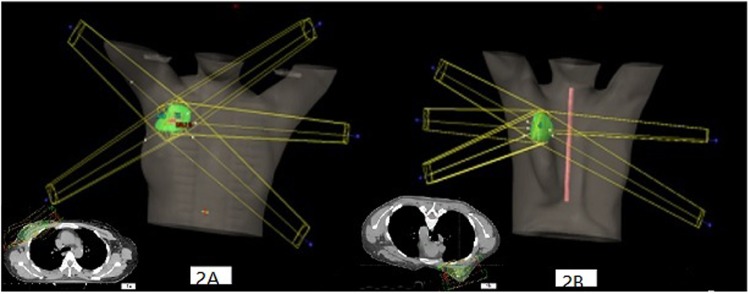
The picture of segmented fields in supine and prone EB-PBI plans (**A**. supine position; **B**. prone position).

The prone and supine EB-PBI plans were both generated in VARIAN’s ECLIPSE TPS Version 13.5 (Anisotropic Analytical Algorithm calculation model). Both plans were generated to deliver a 34-Gy prescription dose given in 10 fractions. The entire treatment process was twice daily and continued 5 days. In addition, the criteria of the plans were to ensure that at least 95% of the PTV received the prescription dose.Three-dimensional conformal radiotherapy (3DCRT) with 6-MV photons in a 4-field noncoplanar beam arrangement was employed in both supine and prone treatment plans. While in supine treatment plan, all the fields were obtained by two tangential fields that were each rotated by 30 degrees and 330 degrees (Fig. 2A), respectively.To reduce the IPSL and ipsilateral breast volume within the supine treatment field as much as possible, 1–2 additional segmented fields were set up to adjust the homogeneity of the target volume (16 patients for one additional segmented field and 14 patients for two additional segmented fields). While in the prone treatment plan, four non-coplanar fields were designed, which were the same as those in the supine treatment plan and 1–2 additional segmented fields were set up to adjust the homogeneity of the target volume (3 patients for one additional segmented field and 27 patients for two additional segmented fields), but the employed noncoplanar beam arrangement increased the ipsilateral irradiated breast volume. Therefore, to reduce the ipsilateral irradiated breast volume and improve the conformity of the target volume, one additional segmented field was added to the prone treatment plan (Fig. 2B).

### Dosimetric evaluation

Target volumes, including the GTV, CTV, PTV and ipsilateral normal breast, were calculated separately for each treatment plan. The distance between the centre of the GTV and the chest wall (*D*_G-c_) was measured for both the prone and supine positions.

Dose-volume histogram (DVH) parameters for the PTV, heart, IPSL, and bilateral breasts were calculated for each plan in all patients. The conformal index (CI) and homogeneity index (HI) were evaluated for the PTV.

CI was defined as follows:

1 $${\rm{CI}}=\frac{{\rm{Ref}}.\,{\rm{isodose}}\,{\rm{volume}}\,{\rm{of}}\,{\rm{the}}\,{\rm{PTV}}}{{\rm{PTV}}}\times \frac{{\rm{Ref}}.\,\mathrm{isodose}\,\mathrm{volume}\,\mathrm{of}\,\mathrm{the}\,\mathrm{PTV}}{{\rm{Ref}}.\,{\rm{isodose}}\,{\rm{volume}}.\,}$$

where Ref.isodose volume of the PTV represents the PTV that is covered by the prescribed dose and Ref.isodose volume is defined as the volume enclosed by the prescribed isodose^14^.

HI was defined as follows:

2 $${\rm{HI}}=\frac{{D}_{2 \% }-{D}_{98 \% }}{{D}_{50 \% }}$$

where *D*_2%_, *D*_98%_ and *D*_50%_ represent the doses covering 2%, 98% and 50% of the PTV, respectively^15^. The IPSL and ipsilateral breast were evaluated using the mean dose (*D*_mean_) and the volumes that received ≥5 Gy, 10 Gy, and 20 Gy (*V*_5_, *V*_10_, and *V*_20,_ respectively). The heart was evaluated using the *D*_mean_, *V*_5_ and *V*_10_. The contralateral breast was evaluated using the *D*_mean_ and *V*_5_.

### Statistical analyses

Statistical analysis was performed with SPSS 19.0 software (IBM Corporation, Armonk, NY, USA). The data that did not follow a normal distribution were described using medians and ranges. The data following a normal distribution was described using average ± standard deviation. The Wilcoxon signed-rank test was used to compare the target volumes and dosimetric parameters. A paired test was used for the comparison of CIs. Spearman rank correlation analysis was performed to establish the relevance between the target volume and exposure dose of the OARs. Data were considered statistically significant at *P* < 0.05.

## Results

### Patient characteristics

Thirty early-stage breast cancer patients who had undergone BCS and were potentially eligible for EB-PBI were enrolled in this study. The median age was 51 (ranging from 38 to 69). Fourteen of the 30 patients had left-sided breast cancer, and the remaining sixteen had right-sided breast cancer. Patients underwent lumpectomy with sentinel lymph node dissection (SLND) or axillary lymph node dissection (ALND) and had ensured tumour-negative margins during a single operation. More than 5 surgical clips (2 mm in diameter) were used to mark the boundaries of the lumpectomy cavity. The surgical clips were placed in the cranial, caudal, medial, lateral, and dorsal walls of the surgical cavity. In addition, the enrolled patients had a seroma clarity score less than 3. The patient and tumour characteristics are presented in Table 1.

**Table 1:** Patient and tumour characteristics.

Variables	Values
**Age, years**	
Median	51
Range	38–69
**Breast side**	
Left	14
Right	16
**Localization of tumour bed**	
UOQ (Left/Right)	17 (8/9)
LOQ (Left/Right)	3 (3/0)
Central portion of breast (Left/Right)	2 (0/2)
UIQ (Left/Right)	5 (2/3)
LIQ (Left/Right)	3 (1/2)
**Tumour characteristics**	
Ductal carcinoma *in situ*	5
Invasive ductal carcinoma	20
Invasive lobular carcinoma	1
Cribriform carcinoma	2
Mucinous carcinoma	2

Abbreviations: UOQ = upper outer quadrant, LOQ = lower outer quadrant, UIQ = upper inner quadrant, LIQ = lower inner quadrant.

### Comparison of the target volume

The GTVs were 14.40 cm^3^ and 14.10 cm^3^ in the supine and prone positions, respectively, with no statistically significant difference (*Z* = −1.40, *P* = 0.162). The CTV and PTV were 57.35 cm^3^ and 108.85 cm^3^ in the supine position, respectively, and 62.60 cm^3^ and 113.7 cm^3^ in the prone position, respectively. After GTV expansion with the described margins, the target volumes, including CTV and PTV, were both significantly higher in the prone position than in the supine position (*Z* = −3.01, −2.87; *P* = 0.003, 0.004, respectively). The ipsilateral breast volume was 31.85 cm^3^ smaller in the supine position than in the prone position (578.00 cm^3^ vs. 609.85 cm^3^). The ratio of the PTV to the ipsilateral breast volume showed no statistically significant differences between the supine and prone positions (*Z* = −0.11, *P* = 0.910), being 17.50% and 18.30%, respectively.

### Comparison of dosimetric parameters

The Wilcoxon signed-rank test showed no significant differences in the PTV coverage of the 100% isodose line (*V*_100%_), which was 97.40% vs. 96.90% for the supine vs. prone positions, respectively (*Z* = −1.752, *P* = 0.08). The CI was 0.69 ± 0.04 and 0.78 ± 0.04 in the supine and prone positions, respectively, with a significant difference (*t* = 9.034, *P* = 0.000). Unlike CI, the HI increased slightly in the prone position relative to the supine position (0.10 vs 0.09, *Z* = −3.137, *P* = 0.002).

Table 2 shows the specified doses for the bilateral breasts, IPSL and heart for the supine and prone treatment plans and shows the comparisons between these two arms, with the *P* values. In our study, no significant differences in the *D*_mean_ to the bilateral breasts were evident between the supine and prone positions. However, for the IPSL, the *D*_mean_, *V*_5_, *V*_10_, and *V*_20_ obtained using the prone treatment plan were all significantly lower than those using the supine treatment plan. The *D*_mean_ to the heart showed a 0.95 Gy reduction in the supine position relative to in the prone position (*P* = 0.000). In addition, a significant difference was also found between the supine and prone treatment plans regarding the *V*_5_ received by the heart. When the tumour bed was in the left breast, a lower dose to the heart was achieved in the supine treatment plan compared with the prone treatment plan. While the tumour bed was in the right breast, a significant difference was also found in the dose to the heart between the prone and supine treatment plans. Moreover, there were no differences found in the *D*_mean_ or *V*_5_ to the heart between left breast cancer patients and right breast cancer patients in supine position (Z = −1.538, −1.245; 0.124, 0.213, respectively). Meanwhile, no significant differences was evident in the *D*_mean_ or *V*_5_ to the heart between left breast cancer patients and right breast cancer patients in prone position (Z = −1.439, −1.287; 0.292, 0.198, respectively).

**Table 2:** Dosimetric evaluation for the supine and prone treatment plans.

Parameters	Supine	Prone	Z	P-value
**Ipsilateral breast**				
D_mean_(Gy)	10.01 (4.49–16.04)	10.40 (4.53–19.59)	−0.70	0.480
**Contralateral breast**				
D_mean_ (Gy)	0.03 (0.00–1.40)	0.05 (0.00–0.98)	−0.32	0.750
**Ipsilateral lung**				
D_mean_ (Gy)	1.72 (0.60–4.27)	1.59 (0.31–4.31)	−2.18	0.029
V_5_ (%)	0.10 (0.02–0.23)	0.07 (0.00–0.44)	−2.17	0.030
V_10_ (%)	0.04 (0.01–0.12)	0.01 (0.00–0.10)	−4.74	0.000
V_20_ (%)	0.01 (0.00–0.07)	0.00 (0.00–0.03)	−4.50	0.000
**Heart**				
D_mean_ (Gy)	0.34 (0.08–2.07)	1.19 (0.04–3.84)	−4.12	0.000
V_5_ (%)	0.00 (0.00–0.18)	0.05 (0.00–0.33)	−3.94	0.000
**Left breast cancer**				
D_mean_-Heart (Gy)	0.47 (0.15–2.07)	2.00 (0.31–3.49)	−3.30	0.001
V_5_-Heart (%)	0.01 (0.00–0.18)	0.09 (0.00–0.33)	−3.11	0.002
**Right breast cancer**				
D_mean_-Heart (Gy)	0.20 (0.08–1.89)	1.13 (0.15–3.84)	−2.53	0.011
V_5_-Heart (%)	0.00 (0.00–0.08)	0.02 (0.00–0.24)	−2.40	0.016

Notes: Data are the median (minimum – maximum) as calculated by SPSS19.0 software. *P* values were calculated with the Friedman test.

Abbreviations: OAR, organs at risk; D_mean_, mean dose (Gy); V_5_, the volumes that received ≥5 Gy; V_10_, the volumes that received ≥10 Gy; V_20_, the volumes that received ≥20 Gy.

### Factors related to OARs and volume change

In both the supine and prone positions, the ratio of the PTV to the volume of the treated breast were well correlated with the *D*_mean_ of the ipsilateral breast (*r* = 0.732, 0.559, *P* = 0.000, 0.010). The *D*_G-c_ was relatively larger in the prone position compared to the prone position (*Z* = −4.328, *P* = 0.000), and the median supine and prone values were 1.25 cm and 1.78 cm, respectively. A statistically significant inverse correlation was found between the *D*_G-c_ and *V*_10_, *V*_20_ and *V*_30_ of the IPSL in the supine and prone positions. Table 3 shows the correlation between the target volume and dosimetric parameters in the supine and prone positions.

**Table 3:** Correlation between the dosimetric parameters of the target volume and OARs.

Parameters	*r*	*P*-value
**Supine**		
IPSB-HI	0.055	0.771
IPSB-CI	0.031	0.394
V_100%_-HI	−0.835	0.000
V_100%_-CI	0.998	0.000
D_G-c_-IPSL V_10_	−0.475	0.008
D_G-c_-IPSL V_20_	−0.739	0.000
D_G-c_-IPSL V_30_	−0.746	0.000
**Prone**		
IPSB-HI	−0.144	0.449
IPSB-CI	0.271	0.148
V_100%_-HI	−0.709	0.000
V_100%_-CI	0.999	0.000
D_G-c_-IPSL V_10_	−0.532	0.002
D_G-c_-IPSL V_20_	−0.831	0.000
D_G-c_-IPSL V_30_	−0.666	0.000

Abbreviations: V_100%_, the PTV coverage of the 100% isodose line; IPSB, the ipsilateral breast; IPSL, the ipsilateral lung; HI, homogeneity index; CI, conformal index; D_G-c_, the distance between the GTV and the chest wall; V_5_, the volumes that received ≥5 Gy; V_10_, the volumes that received ≥10 Gy; V_20_, the volumes that received ≥20 Gy.

## Discussion

Our study demonstrated that the whole breast volume in the supine position was smaller than in the prone position by 31.85 cm^3^. Deseyne *et al*.^16^ also confirmed that for patients who underwent WBI, a small but statistically significant difference was evident in the whole breast CTV between the prone and supine positions (57 cc lower in the prone position). However, for patients with small breasts (<750 cm^3^) who underwent radiotherapy in the prone position, no obvious variations in the breast target volume were found between the supine and prone positions^17^. In our study, the enrolled patients with breast volumes less than 750 cm^3^ comprised 63% of all patients. Therefore, the breast volume discrepancy may have resulted in a change in the breast target volume variance between the supine and prone positions after BCS. However, at present, few studies have assessed the diversity of the tumour bed volume (GTV) between the supine and prone positions for breast radiotherapy^18^. In our analysis, we found no differences in the GTV volume between the supine and prone positions. However, Lakosi *et al*.^18^ observed that in 30 European breast cancer patients, the seroma volume was significantly larger in the prone position than in the supine position. This difference might be caused by the different GTV delineation criteria, whereby the seroma moves away from the chest wall due to gravity in the prone position; however, we delineated the GTV only based on the surgical clips. Lakosi *et al*.^18^ reported a significant increase in the CTV and PTV in the prone position that ranged on average from 13 cm^3^ (CTV) to 22 cm^3^ (PTV) compared with these values in the supine position. This observation shows a stunning consistency with our results. When the ipsilateral breast sagged in the prone position, the breast deformed compared to the breast in the supine position. The distance between the margin of the GTV and the skin/pectorales and D_G-c_ varied with the postural change, and then, the distance expanded in a manner that corresponded to the margin and was subsequently adjusted according to the skin surface/lung-chest wall interface to obtain the CTV and PTV. These volumes varied with the body positions. In addition, the minimum distance between the PTV and the chest wall continued to increase throughout the prone positioning^19^.

Controversy exists regarding WBI treatment in the prone position in terms of target volume coverage and the dose distribution in the target volumes^12,13,17^. Bergom *et al*.^12^ and Alonso-Basanta *et al*.^13^ reported that the dose homogeneity in the prone WBI was improved and that the high dose distribution to the target was also reduced accordingly. Moreover, Kim *et al*.^17^ verified that although there was no difference in the HI between the supine and prone positions, yet a significant advantage in the prone position was found in the CI. Breast and regional lymph nodes can be treated with an equal or better dose distribution in the target volumes in the prone position than in the supine position. For patients treated with WBI, target volume coverage differed only slightly between the prone and supine positions^16^. To the best of our knowledge, our study on the comparison of dosimetric parameters between the prone and supine positions is the first the address the topic using 3DCRT EB-PBI; moreover, the dose distribution discrepancy correlations have also not been investigated previously. We showed that, for Chinese early-stage breast cancer patients, the CI in the prone position was significantly better than that in the supine position. Takahashi *et al*.^20^ reported that the HI was significantly lower in the prone position than in the supine position for patients with large glands. However, for patients treated with EB-PBI, we verified that the variance of the HI between the supine and prone positions was minimal (0.09 vs 0.10) and this difference had nothing to do with the breast volumes (Table 3). Hence, for Chinese patients after BCT, carrying out prone EB-PBI is feasible, and prone EB-PBI has potential advantages regarding the dose distribution in the target volume.

Our results indicated that the primary advantage of prone EB-PBI was the significantly reduced radiation exposure of the IPSL (*D*_mean_, *V*_5_, *V*_10_, and *V*_20_). The *D*_mean_ to the lung met ideal dose constraints in both positions; however, doses, including the *D*_mean_ and *V*_20_, were also significantly reduced in the prone position for IPSL in WBI plan^8,16,19,21,22^. Fernández-Lizarbe *et al*.^21^ found that the prone position reduced the *V*_20_ to the IPSL from 26.5% to 2.9% compared with the *V*_20_ in supine position. Furthermore, in our study, the Spearman rank correlation demonstrated that the *V*_20_ was inversely associated with the *D*_G-c_ for EB-PBI in the prone position and in the supine position (Table 3). Kylie *et al*.^19^ reported that the minimum distance between the seroma cavity PTV and the chest wall was increased with prone positioning (supine, 1.1 mm; prone, 8.7 mm). Thus, a remarkable increase in the distance was evident between the target volumes that were delineated on both the seroma and the surgical clips and the chest wall due to the gland deformation after the postural change, and consequently, the irradiated dose to the lung was reduced.

Lymberis *et al*.^8^ suggested that prone positioning reduced the in-field heart volume in the majority (87%) of left-sided breast cancer patients. However, Würschmidt *et al*.^23^ indicated that no differences were found in the *D*_mean_ to the heart in left WBI between the prone and supine positions. In addition, the *D*_mean_ to the heart in right WBI showed a 0.2 Gy reduction in the supine position relative to in the prone position. As shown in the study by Kirby *et al*.^10^, the irradiated dose to the heart and the left coronary artery in two-thirds of breast cancer patients could be decreased efficiently by prone irradiation. However, by further analysing the correlation, the authors found that only a whole breast CTV >1000 cm^3^ was associated with improved cardiac dosimetry in the prone position. In addition, this has also been confirmed as suitable for partial breast radiotherapy. In our study, only 16.7% of the women had breast volumes >1000 cm^3^, and the heart doses were lower in the supine position than in the prone position. However, Kylie *et al*.^19^ reported that the heart volume was smaller in the prone position than in supine position. To formulate the radiotherapy plan reasonably, one additional segmented field was used to protect the ipsilateral breast, and this field also resulted in the heart dose increasing in the prone irradiation. However, in our study, the *D*_mean_ to the heart was <1.2 Gy in both positions, and a 0.95 Gy reduction in the *D*_mean_ to the heart in the supine position relative to in the prone position still existed. The treatment volume of EB-PBI was smaller than that of WBI, and thus, a smaller irradiation dose was delivered to the normal tissues excluded the PTV. During irradiation in prone EB-PBI, close attention should be paid to the dose delivered to the heart. Currently, Cristoforo *et al*.^24^ confirmed that the application of deep inspiration breath-hold (DIBH) could effectively reduce the mean heart doses by 35% for left-sided breast cancer as compared to free breathing (FB) and appropriately prolong the mean expected years of life after radiation. Meanwhile, significant differences were noted for the mean dose to the heart, left ventricle and left anterior descending artery between supine FB and supine DIBH (SDIBH) in left-breast irradiation, with reduction by 0.84 Gy, 1.69 Gy and 15.43 Gy in SDIBH, respectively^25^. Similarly, Thomas *et al*.^26^ also found that prone DIBH nearly consistently reduced mean heart dose to less then 2 Gy in left-sided whole breast irradiation, regardless of breast volume. This might be attributed to the fact that the application of DIBH could increase the distance between the heart and the chest wall, thus in favor of cardiac protection. It seems that DIBH might be an effective way to reduce the irradiated heart volume and dose, but it is worth noting that the above studies were performed only for left-sided WBI. Meanwhile, prone DIBH needs more time consuming and burdensome due to the extra CT-scan during simulation, treatment plans in DIBH, a longer setup verification procedure with kV-imaging in DIBH and longer treatment time. In addition, prone DIBH puts higher demands on patient compliance. Hence, further research should be done in order to comfirm the feasibility and effectiveness of DIBH in the prone EB-PBI.

The whole breast can be treated with an equal or lower dose distribution to the contralateral breast in the prone position than in the supine position^16,27^. Varga *et al*.^28^ reported that the *D*_mean_ and *V*_5_ of the contralateral breast showed no significant differences between the two positions. However, Verhoeven *et al*.^22^ demonstrated that the *V*_5_ of the contralateral breast was significantly lower in the supine position than in the prone position during either free breathing or a deep inspiration breath-hold in whole breast irradiation therapy. At present, there was no relevant studies to report the effect of respiratory control on the OARs of EB-PBI in the prone position. And in our study, we showed a similar incidental dose to the ipsilateral breast and contralateral breast between the supine and prone 3DCRT EB-PBI plans during free breathing, with an increased incidental dose to the heart in the prone position to avoid increasing the irradiated dose to the contralateral breast. In addition, the *D*_mean_ to the ipsilateral breast was well correlated with the ratio of the PTV to the volume of the treated breast both in the prone and supine positions. Therefore, it was necessary to clarify the patients who would obtain a reduced irradiation dose to the contralateral breast and heart from prone position EB-PBI.

## Conclusions

Although the *D*_mean_ to the OARs met ideal dose constraints in both the prone and supine positions when patients underwent EB-PBI, the heart was still best spared in the supine position. In addition, 3DCRT EB-PBI treatment showed a better dose conformance to the treatment target and a lower dose to the lung in the prone position than in the supine position. Therefore, for the female Chinese patients with early-stage breast cancer, carrying out 3DCRT EB-PBI in the prone position during free breathing was feasible. In the future, further studies on increasing the potential benefits to the heart for prone EB-PBI and on screening patients who are more suitable for prone EB-PBI will be necessary.
